# Loss of CD47 alters CD8+ T cell activation *in vitro* and immunodynamics in mice

**DOI:** 10.1080/2162402X.2022.2111909

**Published:** 2022-09-06

**Authors:** Pulak R. Nath, Dipasmita Pal-Nath, Sukhbir Kaur, Arunkumar Gangaplara, Thomas J. Meyer, Margaret C Cam, David D. Roberts

**Affiliations:** a Laboratory of Pathology, Center for Cancer Research, National Cancer Institute, National Institutes of Health, Bethesda, MD, USA; b Clinical and Translational Immunology Unit, Laboratory of Immunology, National Eye Institute, National Institutes of Health, Bethesda, MD, USA; c Laboratory of Immunology, National Institute of Allergy and Infectious Diseases, National Institutes of Health, Bethesda, MD, USA; d CCR Collaborative Bioinformatics Resource, Office of Science and Technology Resources, National Cancer Institute, Bethesda, MD, USA

**Keywords:** CD47, cytotoxic T cells, acute and chronic T cell activation, lymphocytic choriomeningitis virus, melanoma, RNA-seq, immunotherapy, TCR-crosslinking, thrombospondin-1

## Abstract

CD47 has established roles in the immune system for regulating macrophage phagocytosis and lymphocyte activation, with growing evidence of its cell-intrinsic regulatory roles in natural killer and CD8+ T cells. CD47 limits antigen-dependent cytotoxic activities of human and murine CD8+ T cells, but its role in T cell activation kinetics remains unclear. Using *in vitro* and *in vivo* models, we show here that CD47 differentially regulates CD8+ T cell responses to short- versus long-term activation. Although CD47 was not required for T cell development in mice and early activation *in vitro*, short-term stimuli elevated pathogen-reactive gene expression and enhanced proliferation and the effector phenotypes of *Cd47-*deficient relative to *Cd47-*sufficient CD8+ T cells. In contrast, persistent TCR stimulation limited the effector phenotypes of *Cd47*^−/−^ CD8+ T cells and enhanced their apoptosis signature. CD8+ T cell expansion and activation *in vivo* induced by acute lymphocytic choriomeningitis virus (LCMV) infection did not differ in the absence of CD47. However, the frequency and effector phenotypes of *Cd47^−/−^* CD8+ T cells were constrained in chronic LCMV-infected as well as in mice bearing B16 melanoma tumors. Therefore, CD47 regulates CD8+ T cell activation, proliferation, and fitness in a context-dependent manner.

## Introduction

CD47 is a transmembrane glycoprotein that is ubiquitously expressed in different organs and tissues.^1,2^ In the human immune system, CD47 interacts with some integrins, two counter-receptor signal regulator protein (SIRP) family members, and secreted thrombospondin-1 (TSP1).^1,3–5^ CD47 has two established roles in the immune system. The CD47–SIRPα interaction was identified as a critical innate immune checkpoint, which delivers an antiphagocytic signal to macrophages and inhibits neutrophil cytotoxicity.^6^ CD47 interaction with inhibitory SIRPα is a physiological anti-phagocytic “don’t eat me” signal on circulating red blood cells that is co-opted by some cancer cells.^3,7^ Many malignant cells overexpress CD47.^8–13^ CD47/SIRPα-targeted therapeutics have been developed to overcome this innate immune checkpoint for cancer treatment.^7,9,11,12^ Second, engagement of CD47 on T cells by TSP1 regulates their differentiation and survival^14,15^ and inhibits T cell receptor signaling and antigen presentation by dendritic cells (DCs).^16–21^ CD47 signaling regulates metabolism and mitochondrial homeostasis in multiple cell types,^22–25^ including regulation of global metabolic responses to stress and activation-induced H_2_S signaling in T cells.^17,26^ TSP1/CD47 signaling has similar inhibitory functions to limit natural killer (NK) cell activation^27–30^ and IL1β production by macrophages.^31^ CD47 is, therefore, a checkpoint that regulates both innate and adaptive immunity. The recent understanding of CD47 antagonism associated with increased antigen presentation by DCs^32^ and NK cell cytotoxicity^29^ contributes to the heightened interest in CD47 as a therapeutic target.^33^

Cytotoxic CD8+ T cells are activated in an antigen-specific manner. In a consequence of dynamic TCR-peptidyl-MHC class 1 crosstalk together with co-stimulation and cytokine response, naive CD8+ T cells differentiate into effector CD8+ T cells (Teffs) that undergo robust proliferation and clonal expansion. Differentiated Teffs undergo transcriptional, epigenetic, and metabolic reprogramming and produce effector cytokines and cytotoxic molecules. Teffs induce cytolysis of infected or transformed cells in an antigen-specific manner. The eventual fate of Teffs is contraction, a phase defined by the death of the majority of activated T cells. CD8+ T cell contraction is hardwired and occurs independent of pathogen clearance.^34^ A small group of cells evade contraction and differentiate into memory T cells (Tmems). The hallmarks of Tmems are downregulation of their effector program and acquisition of a stem cell-like property, antigen-independent long survival, and homeostatic self-renewal driven by IL-7 receptor (CD127).^35^

The duration of antigen presentation defines the magnitude of a CD8+ T cell response to a pathogenic infection. This process is fine-tuned in multi-step processes, and expression of their co-signaling receptors play a major role in each step. T cells bear surface receptors that either positively (costimulatory) or negatively (coinhibitory) drive T cell activation or inhibitory signals.^36^ For example, CD28:B7 is a well-studied co-stimulatory axis that amplifies activation signals generated from the TCR-CD3 complex. Other co-stimulatory receptors include ICOS, CD226, OX-40, 4–1BB, and GITR.^36^ Conversely, multiple co-inhibitory T cell receptors can be upregulated, including CTLA4, PD-1, TIM-3, TIGIT, and LAG3. Co-inhibitory receptors control the magnitude of T cell activation, cell-cell contact-mediated immunoregulation, and T cell exhaustion. High expression of co-inhibitory receptors, e.g., PD1, is a hallmark of exhausted T cells,^37^ and blockade of PD1 increases the function of exhausted CD8+ T cells.^38,39^ Interestingly, CD47 has also been found to be upregulated as an early host response to infection, and mice treated with anti-CD47 monoclonal antibody showed enhanced DC activation and CD8+ T cell response to chronic viral infection.^40^ Therefore, understanding the role of CD47 in CD8+ T cell activation, proliferation, and exhaustion is important for designing CD47-targeted immunotherapeutics.

Here we have examined the effect of CD47 on CD8+ T cell stimulation. We have applied short and long TCR-CD3 stimulation of CD47-sufficient and CD47-deficient CD8+ T cells *in vitro* and tested CD8+ T cell physiology utilizing *in vivo* viral infection as well as tumor implantation models in CD47-sufficient and CD47-deficient mice. Short-term TCR stimulation resulted in comparable elevation of gene expression between CD47-sufficient and CD47-deficient CD8+ T cells. However, the molecular profile of CD47-deficient activated CD8+ T cells broadly overlap with pathogen-reactive CD8+ T cells.^41^ Short-term CD3 plus CD28 crosslinking enhanced proliferation and the effector phenotypes of *Cd47^−/−^* CD8+ T cells as evident by the increase of both the gene and protein expression of CD69 as well as elevated interferon gamma response. However, persistent TCR-stimulation limited the ability of *Cd47*^−/−^ CD8+ T cells to produce the effector molecules IFNγ and GzmB. An enrichment of apoptosis signature genes was observed in short-stimulated *Cd47*^−/−^ CD8+ T cells, which resulted in elevated apoptosis after TCR stimulation. Correspondingly, chronic LCMV Cl13 infection as well as implantation of syngeneic B16 melanoma significantly elevated viral titer and also tumor growth, respectively, in *Cd47*^−/−^ mice. Such elevation was correlated with a significant reduction of CD8+ T cells in spleens and within the tumors of *Cd47*^−/−^ mice. Moreover, the effector phenotypes of virus-specific as well as the tumor-infiltrating CD8+ T cells significantly decreased in *Cd47*^−/−^ mice. The regulation of CD47 on CD8+ T cell phenotype and function is context-dependent, as evident by the distinct activation patterns of splenic and tumor-infiltrating CD8+ T cells of the same animal. Taken together, our data suggest that CD47 regulates CD8+ T cell activation, proliferation, and fitness in response to short- and long-term stimuli.

## Materials and methods

### Ethics statement

All animal experiments were carried out in strict accordance with the recommendations for the Care and Use of Laboratory Animals of the National Institutes of Health. The protocols were approved by the NCI Animal Care and Use Committee (Protocol No: NCI/LP-012) and by the National Institute of Allergy and Infectious Diseases Animal Care and Use Committee (Protocol No: LI-5E).

### Mice

Breeding pairs of WT and B6.129S7-Cd47^tm1Fpl^/J (*Cd47*^−/−^) C57BL/6 J mice were purchased from the Jackson Laboratory. *Cd47*^−/−^ mice were backcrossed to minimize genetic drift. Mice were maintained and bred under specific pathogen-free conditions under protocol NCI/LP-012. Littermate and sex-matched mice were used between 6 and 12 weeks of age for experiments. LCMV infection of mice was done under NIAID/LI-5E protocol.

### Reagents

4^′^,6-Diamidino-2-phenylindole (DAPI) (Cat#D9542), rat serum (Cat#R9759), and rabbit serum (Cat#R9133) were purchased from Sigma-Aldrich. Aqua live/dead was from ThermoFisher Scientific (Cat#L3495), UV Zombie was from BioLegend (Cat#423107), and Ammonium-Chloride-Potassium (ACK) lysis buffer was from Lonza (Cat#10-548E).

### Tissue Processing

For preparing single cell suspension, the thymus and spleen were cut into small pieces and mechanically disrupted and flushed out in complete RPMI. Red blood cells (RBCs) were lysed using ACK buffer, and cells were resuspended in FACS buffer. Liver and lungs were cut into small pieces and enzymatically dissociated with Collagenase/Dispase (Roche, Cat# 269638, final concentration 1 mg/ml) and DNase 1 (Sigma, Cat# D4527, final concentration 100 μg/ml). Cells were then filtered through a 70 μm strainer.

### Pan T cell and CD8 T cell isolation and activation

A total peripheral T cell population was isolated from mouse spleens by negative selection using Pan T cell Isolation Kit II (MACS, Miltenyi Biotec) to deplete cells expressing CD11b, CD11c, CD19, CD45R, CD49b, CD105, MHC class II, and Ter-119. CD8^+^ cells from the isolated Pan T cells were further sorted using FACSAria II (BD Biosciences). Total CD8+ T cells and untouched naïve CD8+ T cells from WT and CD47 null mice were extracted using the mouse CD8a+ T Cell Isolation Kit (Order no. 130–104-075) and mouse Naive CD8a+ T Cell Isolation Kit (Order no. 130–096-543), respectively, from Miltenyi Biotec according to the manufacturer’s instructions. Purified Hamster Anti-Mouse CD28 (BD Pharmingen™) and CD3 Monoclonal Antibody (17A2), Functional Grade (eBioscience™), were used to 12 well plate (Corning, USA) coated with anti-CD3 Antibody (2 μg/ml) for overnight at 4°C, rinsed with sterile PBS1x next day and plated with freshly isolated CD8+ T cells (mouse CD8a+ T Cell Isolation Kit, Miltenyi Biotec) in RPMI 1640 medium with 10% FBS. Soluble anti-CD28 antibody (4 μg/ml) was added to the cell suspension for the indicated time. For bulk RNAseq analysis, freshly isolated CD8+ T cells were stimulated by plating on anti-CD3 + anti-CD28-coated plates for 6 h in RPMI 1640 medium with 2% FBS. The untreated CD8^+^ T cells were used as control.

### Cell proliferation analysis by flow cytometry

Isolated cells were pulsed with 5 μM carboxyfluorescein diacetate succinimidyl ester (CFSE) for 30 min at room temperature protected from light. Cells were then washed and cultured in complete RPMI with TCR stimuli. Cell proliferation was followed for 2 days. Cells were acquired using a BD LSR Fortessa SORP Flow Cytometer with 355-nm excitation and a 450/50-nm bandpass emission filter. The discrete peaks in the histograms represent successive generations of live T cells. An overlay of the unstimulated parent generation is indicated as the brightest peak on the far right side of the histograms.

### Apoptosis analysis

Cells were stained with annexin V according to the manufacturer’s instructions (BD Biosciences, cat. #550474). Cells were washed in PBS and resuspended in 1 annexin binding buffer containing allophycocyanin-conjugated annexin V. After 15 minutes of incubation at room temperature, cells were diluted in 1 annexin V binding buffer and analyzed by flow cytometry.

### LCMV infection and plaque assay

LCMV Cl-13 viruses (Dr. Ethan Shevach, NIAID) were propagated in baby hamster kidney-21 fibroblast cells [American Type Culture Collection (ATCC), Manassas, VA, United States]. Viral titers were determined by plaque assay using Vero African-green-monkey kidney cells (ATCC), as described elsewhere.^38^ Viral stocks were frozen at −80°C until used. Mice were infected with the diluted virus in 1x sterile phosphate buffer saline (PBS) (Armstrong virus, 2 × 10^5^ plaque forming unit (pfu)/mouse, i.p., or Cl-13 virus, 2 × 10^6^ pfu/mouse, i.v.).

### Tumor implantation

Banked cryopreserved B16F10 melanoma cells (obtained from ATCC in 2007) were defrosted and cultured in a T-75 flask for 48 hours in complete RPMI-1640 medium containing 5% FBS, 1% penicillin and streptomycin antibiotics, and 1 mmol/L L-glutamine. Because banked cells at identical passage were used for all animal studies, their identity was verified as negative for known murine viruses and Mycoplasma by the Animal Health Diagnostic Laboratory, Frederick National Laboratory. WT or *Cd47^−/−^* littermate C57BL/6 mice were subcutaneously injected with PBS-washed B16F10 melanoma cells (1 x 10^6^ cells) into the hind limb. Tumor size was compared on day 15 at the termination of the experiment.

### Tumor processing

Tumors were cut into small pieces and enzymatically dissociated with Collagenase/Dispase (Roche; cat. #269638; final concentration 1 mg/mL) and DNase 1 (Sigma; cat. #D4527; final concentration 100 mg/mL). The suspension was centrifuged at 50 g for 5 minutes to separate tissue debris, and supernatant cell suspension was filtered through a 70-mm strainer, followed by centrifugation with 330 g for 5 minutes. Red blood cells (RBC) were lysed using ACK buffer, and cells were suspended in FACS buffer followed by staining or sorting.

### RNA extraction, quantitative real-time PCR, and primer sequences

RNA was purified from the indicated cell types using TRIzol following the manufacturer’s instructions. RNA was reverse transcribed to cDNA using Thermo Scientific Maxima First Strand cDNA Synthesis Kit for RT-qPCR. Quantitative real-time PCR was performed with SYBR Green using primers for specific genes and analyzed on CFX96 Real-time System (Bio Rad). Relative transcript abundance was determined by using the ΔΔ*C_t_* or Δ*C_t_* method after normalization with β*-Actin* and *Gapdh*. All samples were run in triplicate. Error bars represent S.E.M.

### Flow cytometry and cell sorting

Single cell suspensions from organs and tissues were prepared as described above. Cell preparations were stained with optimized antibody dilutions. Antibodies used in the lineage cocktail (Lin) include, but not limited to, antibody against B220 (RA3-6B2), CD19 (eBioD3), Gr1 (RB6-8C5), CD11c (N418), and Ter119 (TER-119). Additional antibodies used included those targeting antibody molecules CD45.2 (104), CD8 (53–6.7), CD3 (145–2C11), CD69 (H1.2 F3), CD44 (IM7), CD62L (MEL-14), KLRG1 (2F1/KLRG1), PD1 (29 F.1A12), and CD47 (miap301) and intracellular molecules TNFα (MP6-XT22), Ki-67 (SolA15, B56), IFNγ (XMG1.2), and Granzyme B (NGZB, GB11). Antibodies were directly conjugated to Brilliant Ultraviolet (BUV)395, Brilliant Violet (BV)786, BV711, BV650, BV605, Pacific Blue (PB), fluorescein isothiocyanate (FITC), phycoerythrin (PE), PE-Cy5.5, PE-Texas Red, peridinin-chlorophyll-protein complex (PerCP)-Cy5.5, PE-Cy7, allophycocyanin (APC), or APC-Alexa 700. All antibodies were purchased from either eBioscience/Biolegend/BD Pharmingen. Cells were resuspended in FACS buffer (1% BSA+0.01% NaN_3_ in PBS1x, filtered) and incubated with rat plus rabbit serum followed by incubation with antibody cocktail against surface molecules. For intracellular staining, cells were fixed (IC Fixation Buffer, eBioscience) and permeabilized (Permeabilization Buffer 10x, eBioscience) and incubated with antibodies against intracellular molecules.

Cell sorting was performed on a FACSAria II (BD Biosciences), and flow cytometric analysis was performed on a LSR Fortessa SORP (BD Biosciences). Dead cells were excluded through 4,6 diamidino-2-phenylindole (DAPI) uptake or aqua live/dead staining. Doublets were excluded through forward scatter–height by forward scatter–width and side scatter–height by side scatter–width parameters. Data were analyzed using FlowJo (Tree Star).

### Bulk RNA sequencing: sample preparation and data analysis

Total RNA of untreated and activated CD8+ T cells (n = 4, biological replicates) from WT (*Cd47*^+/+^) and KO (*Cd47*^−/−^) were isolated using the TRIzol RNA extraction protocol. The RNA concentration and purity were determined using Nano-drop and Agilent 2100 Expert bioanalyzer (Agilent Technologies). RNA quality was reported as a score from 1 to 10, and samples falling below the threshold of 8.0 were excluded from the study. RNA sequencing was performed at the core facility of Frederick National Laboratory for Cancer Research (Leidos Biomedical Research, Inc) using HiSeq3000 with Illumina v4 chemistry. Base calling was performed using RTA version 1.18.66.3 and demultiplexing by Bcl2fastq version 2.17 allowing one mismatch. All the mRNA sequenced samples had a yield ranging from 24 to 38 million reads and pass filter reads of 89–94% ≥ Q30.

Samples were trimmed for adapters and low-quality bases using Trimmomatic version 0.36^42^ software and aligned with reference mouse mm10 genome using STAR version 2.5.1^43^ software. Quantification was carried out with RSEM (version 1.2.22)^44^ using the transcriptome .bam files created by STAR. Alignment percentages range from 87 to 96% of reads. RNA mapping statistics are calculated using Picard software. For all the samples, coding bases are around 40–59% and mRNA bases are above 66%. Percent ribosomal bases is below 1% for all samples. Library complexity is measured by unique fragments in the mapped reads using Picard’s Mark Duplicate utility. There were between 58 and 88% non-duplicated reads.

Downstream analysis and visualization were performed within the NIH Integrated Data Analysis Portal (NIDAP) using R programs developed on the Foundry platform (Palantir Technologies). Briefly, raw counts data produced by RSEM were imported into the NIDAP platform, genes were filtered for low cowns (<1 cpm), and the voom algorithm^45^ from the Limma R package (version 3.40.6)^46^ was used for quantile normalization and analysis of differentially expressed genes (DEGs). Genes with adjusted p-value ≤0.001 and log2 fold change of ≥1.0 were considered significantly differentially expressed. Pre-ranked GSEA using the t-statistics derived from the DEG analysis was performed using the Molecular Signatures Database version 6.2^47^ and the GSEA package.^48^ 

The CD47 dependent gene list was extracted using normalized DEGs from contrasts WT_CD3_CD28 vs WT_UT (WT-activated) and CD47KO_CD3_CD28 vs CD47KO_UT (Cd47 null activated). The log fold change was determined using formula log2FC = Log2(Cd47 null activated)-Log2(WT-activated), and fold change was calculated using Power (2, log2_Value) with cutoff of ≥1.5. The comparison of CD47 dependent genes were compared with all genes and gene clusters I, II, VII, and VIII for CD8+ T cell activation^41^ using FunRich-Functional Enrichment Analysis Tool (www.funrich.org).

### Statistical analysis

Graphs were generated and statistical analysis on groups with limited variance was performed using GraphPad Prism 7 (Version 7.01). Comparison between two groups was done via unpaired two-tailed Student’s *t*- test. Differences with a *p *< 0.05 were considered significant.

## Results

### CD47 is dispensable for T cell development

Thymus provides a unique environment where early T-lineage progenitors (ETPs) become committed to differentiate, proliferate, and develop into mature CD4 and CD8 single positive T cells. The ETPs acquire interleukin-2 receptor (CD25) to differentiate to CD4 and CD8 double negative 2 (DN2) stage, marking T-lineage commitment. As the differentiation progresses, DN2 cells undergo somatic rearrangement of their TCR loci by V(D)J recombination and give rise to DN3b1 and DN3b2 cells in a process called β-selection. Massive proliferation and subsequent massive cell death through intensive positive and negative selections occur at the final stage of development, yielding selected mature T cells expressing either CD4 or CD8 markers.

**Figure 1.**
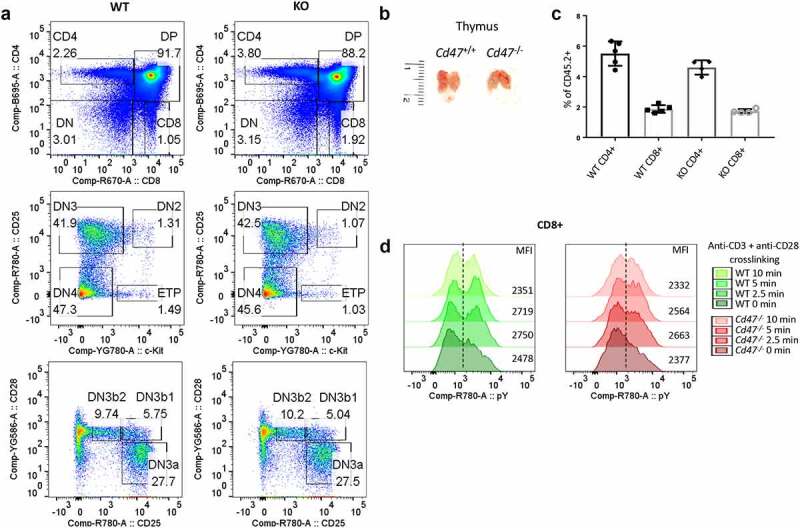
CD47 is dispensable for T cell development.

Several previous studies suggested that CD47 plays an essential role in T cell development, trafficking, and homeostasis. Expression of murine CD47 on human progenitor cells increased the generation of mature CD4+ and CD8+ T cells in engrafted mice.^49^ CD47-dependent integrin regulation also regulates T cell transmigration and survival.^50,51^ However, consistent with a previous report that thymic differentiation of T cells was not impaired in *Cd47^−/−^* mice,^52^ deficiency of CD47 did not alter T cell precursor populations in the thymus (Figure 1a). Frequency and migration of ETPs into the thymus were unchanged in *Cd47^−/−^* mice. The process of T cell development from ETP to DN2 to DN3a, DN3b1, DN3b2, and DN4 stages in *Cd47*^−/−^ thymus was comparable to their WT counterparts. Development of DP cells as well as CD4+ and CD8+ single positive cells is also not affected in the absence of CD47 (Figure 1a,b,c). Consistent with our previous report that TSP1 inhibits TCR signaling in Jurkat T cells downstream of ZAP70 and LAT phosphorylation,^53^ thymocytes activated by engaging TCR-CD3 complex and crosslinking up to 10 minutes exhibited no difference in the global tyrosine phosphorylation level between WT and *Cd47*^−/−^ CD8+ T cells (Figure 1d).

(a) Single cell suspensions from thymus of WT (*Cd47*^+/+^) and KO (*Cd47^−^*^/-^) littermate and sex-matched mice were FcR-blocked and stained for CD45.2, CD3, CD4, CD8, CD25, c-kit, and CD28. DAPI was used to discriminate live/dead cells. Live, CD45.2 cells were gated for CD4 and CD8 expression. CD4-CD8- double negative (DN) cells are further gated for c-Kit, CD25 and CD28 expression to distinguish among early thymic progenitor (ETP), double negative (DN)2, DN3, DN3a, DN3b1, DN3b2, and DN4 population, n = 5. (b) Thymus of 8 weeks old WT and *Cd47*^−/−^ littermate female mice are comparable in sizes, n = 5. (c) Frequencies of CD4+ and CD8+ single positive cells in thymus are comparable between WT and *Cd47^−^*^/-^ mice. n = 5. (d) Pan T cells were isolated from the thymus of WT and *Cd47^−^*^/-^ mice and incubated with anti-CD3 plus anti-CD28 on ice and then on 37°C for the indicated time. Cells were immediately fixed, permeabilized, and intracellular stained for anti-phosphotyrosine (pY). CD8+ T cell subsets were flow gated and analyzed for intracellular pY. Representative of two independent experiments.

### CD47 regulates proliferation and activation of CD8+ T cells

**Figure 2.**
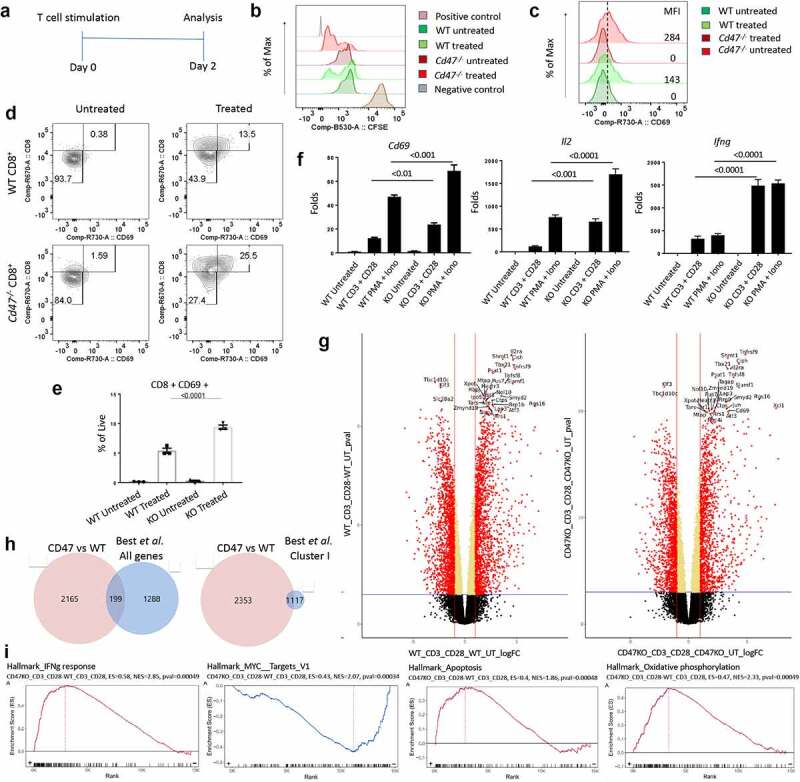
CD47 regulates proliferation and activation of CD8+ T cells.

TSP1 is a CD47-dependent inhibitor of human and murine T cell activation and proliferation,^16,17,53,54^ and splenic CD3+ T cells from *Cd47*^−/−^ and *Thbs1^−/−^* mice consequently exhibit increased acute activation-dependent IL-2 induction.^17^ TSP1/CD47 signaling has a similar inhibitory role in NK cell proliferation and activation^28,29^ and macrophage IL-1β production.^31^ To further evaluate the role of CD47 in CD8 T cell proliferation and activation, we isolated a pan T cell (selected population from spleen negative for CD11b, CD11c, CD19, CD45R, CD49b, CD105, MHC class II, and Ter-119 expression) from WT and *Cd47*^−/−^ mice and pulsed them with CFSE. Cells were then seeded on uncoated plates or plates coated with anti-CD3 (plus soluble anti-CD28) in complete medium (Figure 2a). Gating on the CD3+ CD8+ T cells by flow cytometry, we found that both WT and *Cd47*^−/−^ CD8+ T cells diluted CFSE within 48 hour of TCR stimulation (Figure 2b). However, CFSE dilution was more prominent in the *Cd47*^−/−^ CD8+ T cells, indicating increased proliferation in the absence of CD47. Similarly, mean fluorescence intensity (MFI) of the T cell activation marker CD69 was upregulated in both WT and *Cd47*^−/−^ CD8+ T cells upon stimulation but more in activated *Cd47*^−/−^ than activated WT CD8+ T cells (Figure 2c).

(a) Experimental schematic showing activation and analysis plan. (b) Pan T cells were isolated from the spleens of WT (*Cd47*^+/+^) and *Cd47^−^*^/-^ littermate and sex-matched mice and pulsed with CFSE. Cells were then plated on anti-CD3-coated plate plus anti-CD28 in the media (treated). Forty-eight hours later, dilution of CFSE by CD3+ CD8+ compartment was measured by flow cytometry. Positive control is splenocytes freshly stained with CFSE, and the negative control is unstained splenocytes. Representative of two independent experiments. (c) CD69 expression in the CD3+ CD8+ compartment was analyzed (untreated = media only, treated = anti-CD3 plus anti CD28). Representative of experiments using three mice. (d) CD8+ T cells were isolated from spleens and cultured only in media (Untreated) or with anti-CD3 plus anti-CD28 (Treated) for 48 hours. Cells were then FcR-blocked and stained for CD45.2, CD3, CD8, and CD69. DAPI was used to discriminate live/dead cells. The numbers indicated are the frequency of CD8+ T cells. Representative of three mice. (e) Frequency of CD8+ CD69+ cells after the treatment. Numbers indicated are the frequency of live cells, n = 3. (f) Isolated CD8+ T cells from spleens of WT and *Cd47^−^*^/-^ mice were stimulated with anti-CD3 plus anti-CD28 for 24 hours or PMA plus ionomycin for 6 hours, and relative expression of *Cd69, Il2*, and *Ifng* mRNAs were measured by qPCR. Fold change is relative to expression in WT untreated, n = 3. (g) Analysis of bulk RNAseq for CD8+ T cells isolated from spleens of WT and *Cd47^−^*^/-^ mice with or without activation on immobilized anti-CD3 plus anti-CD28 for 6 h. Volcano plots showing differentially expressed genes (DEGs) of WT_CD3 + CD28 vs WT_Untreated and CD47KO_CD3 + CD28 vs CD47KO_Untreated with a *p*-value threshold ≤0.001 and log_2_ fold-change threshold at least 1.0. The significant DEGs are shown as in red dots, and positive values (right side) are upregulated while negative value (left side) are downregulated genes on the logFC-axis. The top 30 genes are indicated with label of genes names, n = 4. (h) Comparing activation-dependent genes in WT and *Cd47^−^*^/-^ CD8+ T cells identifed 2,364 genes that were ≥1.5-fold differentially regulated in CD47KO over WT. Of these CD47-dependent genes, FunRich gene tools identifed 199 in common with the 1,487 altered gene transcripts and 11 0f the 28 Cluster-I genes in pathogen-reactive CD8+ T cells from Best et al.^41^ . (i) GSEA enrichment using the MSigDB v6.2 (Human/Mouse/Macaque) with default setting of GSEA Visualization (Bulk/CCBR/v362).

To rule out any indirect effect from assessing the activation using pan T cells, we isolated CD8+ T cells from WT and *Cd47*^−/−^ spleens. Cells were cultured similarly on anti-CD3-coated plate (plus soluble anti-CD28) for 48 hours. We observed significantly more expansion of the CD69+ population upon stimulation of *Cd47*^−/−^ compared to the WT CD8+ T cells (Figure 2d,e). To further validate the observation at the mRNA level, we performed gene expression analysis of *Cd69* using qPCR. TCR stimulation for 24 hours or stimulation with PMA-ionomycin for 6 hours both upregulated the gene expression of *Cd69*. Importantly, the relative gene expression of *Cd69*, along with other activation markers *Il2* and *Ifng*, were many folds upregulated in stimulated *Cd47*^−/−^ CD8+ T cells compared to the WT CD8+ T cells (Figure 2f).

## CD47 regulates effector and apoptotic transcriptional pathways in CD8+ T cells responding to early activation

TCR stimulation orchestrates a cascade of signaling events leading to activation and nuclear-translocation of transcription factors that in turn results in increased transcription of effector genes. Based on the involvement of co-stimulatory molecules and duration of the stimulation, the transcriptional signatures can greatly vary. In our earlier work, we have observed CD47 to be a potent regulator of global gene expression in NK cells. While the naïve and memory gene signatures were significantly downregulated, the early, sustained, and late effector gene signatures of NK cells were significantly upregulated in the absence of CD47.^28^ Similarly, thrombospondin-1 signaling via CD47 globally inhibited early anti-CD3 activation of human Jurkat T lymphoma cells and primary mouse T cells,^17,53,54^ and *Cd47*^−/−^ T cells exhibited increased activation in an oxazolone inflammation model.^15,55^ To further define the role of CD47 in regulating the transcriptional profile of CD8+ T cells during the early activation, CD8+ T cells isolated from spleens of WT and *Cd47*^−/−^ mice were cultured for 6 hours on plates coated with anti-CD3 plus anti-CD28. Bulk RNA sequencing revealed a total of 3664 genes in WT CD8+ T cells and 2900 genes in *Cd47*^−/−^ CD8+ T cells to be differentially expressed upon activation. A majority of the differentially expressed genes, 2451 total, including the highly significant *Il2ra, Shmt1, Cish, Tbx21, Tnfsf8, Tnfsf9, and Smalf1* were upregulated in both WT and *Cd47*^−/−^ CD8+ T cells (Figure 2g, Data S1, S2). Expression of *Thbs1*, encoding the CD47 inhibitory CD47 ligand TSP1, did not differ between WT and *Cd47*^−/−^ cells and was not significantly altered following 6 h activation of CD8+ T cells. Next, we filtered 2,364 shared genes that were at least 1.5-fold differentially expressed upon 6 h activation in WT versus *Cd47*^−/−^ CD8+ T cells (Data S3) and compared them with transcriptional profiles identified in pathogen-reactive CD8+ T cells.^41^ Those authors selected 1,487 genes that had a difference in expression of at least twofolds in response to *Listeria monocytogenes* (Lm-OVA) infection and clustered into 10 unbiased groups with the most dynamic patterns according to kinetic patterns of expression (by *K*-means clustering). We found 199 of the early (12 h) activation genes in pathogen-reactive CD8+ T cells identified by Best et al.^41^ to be differentially expressed in WT versus *Cd47*^−/−^ CD8+ T cells after 6 h anti-CD3 plus CD28 stimulation (Figure 2h, Data S4). These included 11 of the 28 genes in Cluster I (Cluster I included genes with expression that was upregulated 12 h after activation, then decreased immediately but remained higher than that in naive cells) of Best et al.^41^ that were induced in Lm-OVA reactive CD8+ T cells 12 h after immunization (Figure 2h, Data S5). These CD47-dependent activation genes included *Cxcl10, Cxcl11, Gzmb, Gzmc, Il2Ra, Irf4, IL21*, and *Il2*. Comprehensive comparison using GSEA showed significant upregulation of interferon gamma response (NES = 2.85, *p* < 0.001), apoptosis (NES = 1.86, *p* < 0.001), and oxidative phosphorylation (NES = 2.33, *p* < 0.001) signature genes in 6 h activated CD47-deficient CD8+ T cells compared to WT CD8+ T cells (Figure 2i). Consistent with the downregulation of Myc in CD8+ but not CD4+ mouse T cells,^4^ a significant downregulation of Myc targets was observed in 6 h activated CD47-deficient compared to WT NK cells (NES = −2.07, *p* < 0.001, Figure 2i).

### TCR stimulation of CD47-deficient CD8 T cells marks elevated cytokine and apoptosis signature simultaneously

**Figure 3.**
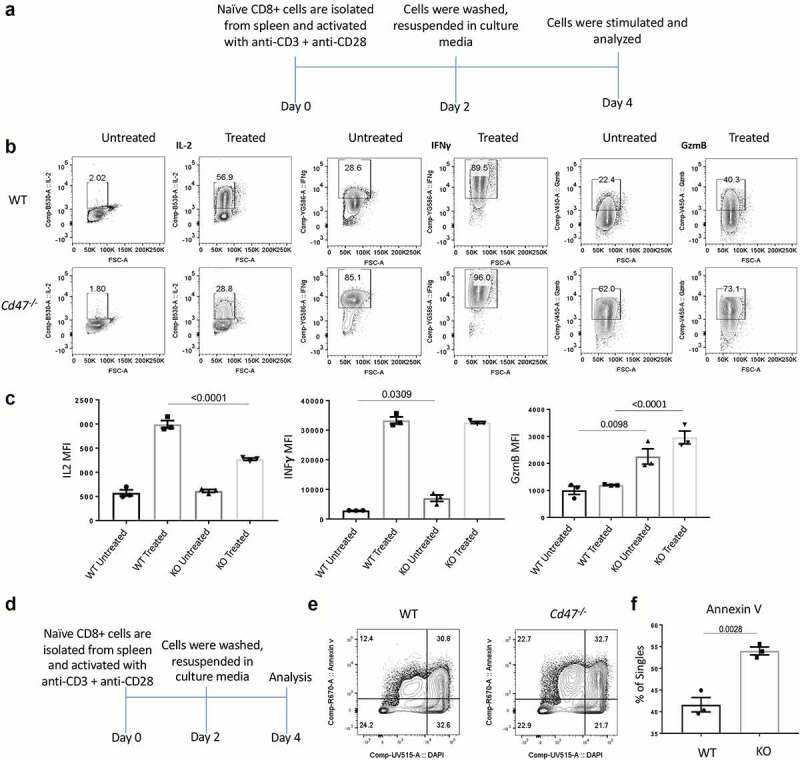
TCR stimulation of CD47-deficient CD8 T cells marks elevated cytokine and apoptosis signature within 4 days of stimulation.

We did not find any major impairment in CD8+ T cell development and initial activation in the absence of CD47. Rather both physiological and pharmacological stimulation elevated activation phenotype of CD47-deficient CD8+ T cells. However, previous studies have identified additional roles for CD47 in T cell contraction and apoptosis after stimulation *in vitro* and *in vivo*.^15,55–58^ To further examine this in an *ex vivo* setting, we isolated naïve CD8+ T cells from WT and *Cd47*^−/−^ mice spleens and pulsed the cells with anti-CD3 (plate-coated) plus anti-CD28 (soluble) antibodies. Forty-eight hours later, the cells were washed with PBS1x and re-plated with complete media for another 48 hours (Figure 3a). Finally, on day 4, cells were restimulated using the lymphocyte activation cocktail with Golgi-stop for 4 hours and stained for intracellular IL-2, interferon gamma (IFNγ), and granzyme B (GzmB) (Figure 3b). We found significantly less IL-2 accumulation (*P* < 0.0001) in late activated *Cd47*^−/−^ CD8+ T cells (Figure 3c). However, accumulation of both IFNγ and GzmB in previously stimulated *Cd47*^−/−^ CD8+ T cells was significantly more (IFNγ, *P* = 0.03; GzmB, *P* = 0.009) than the correspondingly treated WT CD8+ T cells. An additional 4 hours of final activation significantly increased GzmB accumulation (*P* < 0.0001) in *Cd47*^−/−^ CD8+ T, while the accumulation of IFNγ was comparable with that observed in WT CD8+ T cells.

(a) Experimental schematic showing the activation and analysis plan. (b) Naïve CD8+ T cells were isolated from the spleens of WT and *Cd47^−^*^/-^ littermate and sex-matched mice and cultured in complete media only (Untreated) or plated on anti-CD3 coated plates plus anti-CD28 in the media (Treated). Forty-eight hours later, all cells were washed in sterile PBS 1x and cultured in complete media for another 48 hours. On day 4, cells were pulsed with Leukocyte activation cocktail (BD) for 4 hours with Golgi Stop. Cells were FcR-blocked and stained for CD45.2, CD3, and CD8 followed by fixation, permeabilization, and intracellular staining for interleukin-2 (IL-2), interferon gamma (IFNγ), and granzyme-B (GzmB). Representative of two independent experiments, the cells are pooled from three mice for each experiment. (c) Mean fluorescence intensity (MFI) of IL-2, IFNγ, and GzmB expression in the CD3+ CD8+ cells on day 4th after restimulation (untreated = media only, treated = anti-CD3 plus anti-CD28), n = 3. (d) Experimental schematic showing the activation and analysis plan for panels E,F. CD8+ T cells were isolated from spleens and with anti-CD3 plus anti-CD28 and 48 hours later cells were washed and cultured in complete media for another 48 hours. Cells were stained for Annexin V and DAPI. (e, f) Representative plot and frequency of Annexin V and DAPI-positive cells after the treatment, n = 3.

The significant decrease in IL-2 production after restimulation suggested impairment in the growth signaling pathways in *Cd47*^−/−^ CD8+ T cells, which may lead to elevated cell death. To test this hypothesis, we performed a similar experiment as 3A, and on day 4, we analyzed the expression of the apoptosis marker annexin V (Figure 3d). We found significant increase of annexin V+ cells in the *Cd47*^−/−^ CD8+ cells (Figure 3e,f), indicating elevated post-stimulation apoptosis of these cells.

## CD47-deficiency limits the activation state of CD8 T cells upon prolonged TCR stimulation

**Figure 4.**
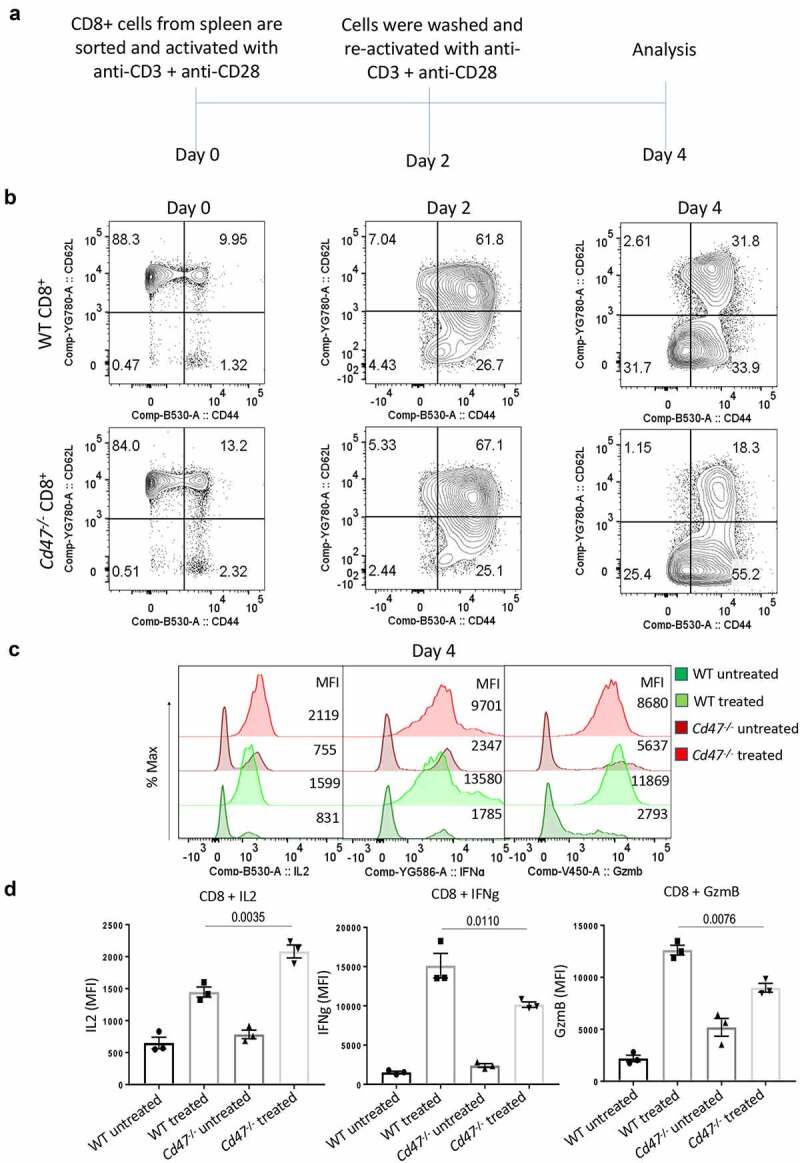
CD47 deficiency limits activation state of CD8 T cells upon prolonged TCR stimulation.

Our data suggested elevated contraction of *Cd47*^−/−^ CD8+ T cells after stimulation. However, the levels of effector proteins IFNγ and GzmB were higher in these cells compared to the WT CD8+ T cells, indicative of elevated activation status. We used restimulated T cells to compare the sustained activation phenotypes of WT and *Cd47*^−/−^ CD8+ T cells. Isolated naïve CD8+ T cells from WT and *Cd47*^−/−^ spleens were stimulated using anti-CD3 (plate-coated) plus anti-CD28 (soluble) antibodies. Cells were washed with PBS1x on the 2nd day and re-stimulated with plate-coated anti-CD3 (plus soluble anti-CD28) antibodies (Figure 4a). On day 4, we added Golgi stop and four hours later stained cells for surface CD44 and CD62L and intracellular IL2, IFNγ and GzmB (Figure 4b). We observed a clear shift of population from naïve (CD44- CD62L+) on day 0 to effector (CD44+ CD62L-) and central memory (CD44+ CD62L+) on day 2 and day 4 upon TCR stimulation. An almost 1.5-fold increase in the CD44+ effector population was evident in *Cd47*^−/−^ CD8+ T cells on day 4. Although the accumulation of intracellular IL2 was significantly higher in *Cd47−/− *CD8 T cells, the accumulation of IFNγ and GzmB levels on day 4 was significantly less in *Cd47*^−/−^ compared to those in WT CD8+ T cells after sustained TCR stimulation (Figure 4c,d). The data clearly suggest that CD47-deficiency impaired the sustained activation phenotype of CD8+ T cells under persistent TCR stimulation.

(a) Experimental schematic showing the activation and analysis plan. Naïve CD8+ T cells were isolated from WT and *Cd47^−^*^/-^ littermate and sex-matched mice and cultured in complete media only (Untreated) or plated on anti-CD3 coated plate plus soluble anti-CD28 (Treated). Forty-eight hours later all cells were washed in sterile PBS 1x and re-plated on freshly coated anti-CD3 plus soluble anti-CD28 in complete media for another 48 hours. Representative of two independent experiments. (b) CD44 and CD62L expression profiles of cells on day 0, day 2, and day 4 of treatment. (c) Representative plots showing intracellular interferon gamma (IFNγ) and granzyme B (GzmB) levels of CD8+ T cells on the 4th day of treatment. (d) MFI of intracellular IFNγ and GzmB levels of CD8+ T cells on the 4th day of treatment, n = 3.

## CD47 deficiency elevates acute LCMV infection in mice

Lymphocytic choriomeningitis virus (LCMV)^59^ infection of mice is the most used model to study the dynamics of CD8+ T cell responses to viral infection. While LCMV-Armstrong (Arm) provoke an acute infection and virus is eliminated within 7-10 days, the LCMV-clone 13 (Cl13) results in chronic infection in mice where the virus actively replicates for months. In response to the persistent infection, a distinct transcriptional signature^60^ and epigenetic modifications^61^ are imprinted in virus-specific CD8+ T cells. We found that the LCMV-Arm is the closest infection model to replicate short-term stimulation of CD8+ T cells *in vivo*, while LCMV-Cl13 is the closet infection model to replicate the persistent TCR stimulation.

**Figure 5.**
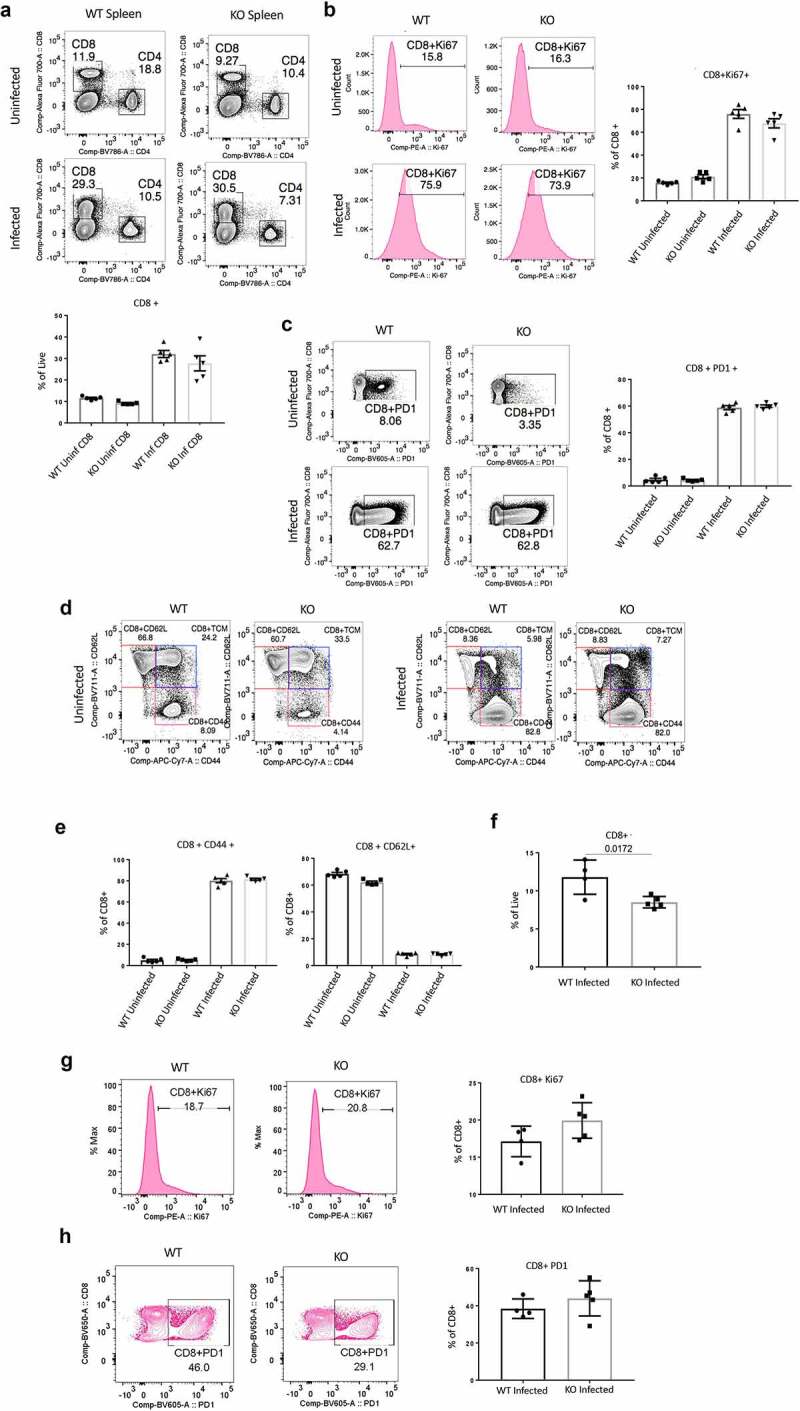
Elevated acute LCMV infection in CD47-deficient mice is associated with impaired CD8+ T cell response.

As we observed in our earlier study, LCMV-Arm viral titer in the serum of *Cd47*^−/−^ mice was significantly high on day 8 post infection.^28^ CD8+ T cells expanded many folds higher in the spleens of infected mice than the uninfected counterparts. However, there was no significant difference between WT and *Cd47*^−/−^ mice (Figure 5a). Similarly, the frequency of Ki67+ CD8+ T cells increased from 20% in uninfected to 80% in infected mice, suggesting extensive cell proliferation in both WT and *Cd47*^−/−^ animals upon viral infection (Figure 5b). Infection-induced upregulation of PD1 in both WT and *Cd47*^−/−^ CD8+ T cells was comparable (Figure 5c). While characterizing the activation status of CD8+ T cell, we found at least a 10-fold increase in the effector phenotype (CD44+) and a concomitant decrease in the naïve phenotype (CD62L+) that was evident on the 8th day of infection and with no apparent difference between WT and *Cd47*^−/−^ mice (Figure 5d,e). These data clearly suggest that deficiency of CD47 does not impact CD8+ T cell early immune dynamics of antigen-recognition, activation, and proliferation at the acute state of infection.

(a) Representative contour plots (values indicate percentage of CD3+ cells) and frequency of CD8+ T cells within the spleens of uninfected and acute LCMV-Armstrong (LCMV-Arm) infected mice on the 8th day post infection. Representative histograms and frequency of Ki-67+ CD8+ T cells (b) and representative counter plots and frequencies of PD1+ CD8+ T cells (c) within the spleens of infected mice on the 8th day post infection. Representative of two independent experiments, n = 5, mice in each group. (d) Representative contour plots (values indicate percentage of CD8+ cells) and (e) frequencies of naïve (CD44-CD62L+), T central memory (Tcm, CD44+ CD62L+) and T effector memory (CD44+ CD62L-) CD8+ T cells within the spleens of uninfected and LCMV-Arm infected mice on 8th day post infection, n = 5. (f) Frequency of CD8+ T cells within the spleens of LCMV-Arm infected mice on the 25th day post infection. Representative histograms and frequency of Ki-67+ CD8+ T cells (g) and representative counter plots and frequency of PD1+ CD8+ T cells (h) within the spleens of infected mice on the 25th day post infection. Representative of two independent experiments, n = 4 WT mice and 5 *Cd47*-/- mice.

With chronic LCMV-Cl13 infection to mice, serum viral titers were significantly higher in *Cd47*^−/−^ mice compared to WT mice on the 25th day of infection.^28^ Interestingly, at this point, the frequency of CD8+ T cells dropped significantly in *Cd47*^−/−^ mice (Figure 5f). The proliferation (Ki67 level) and exhaustion (PD1 level) of CD8+ T cells were constrained at this time point compared to day 8 post infection (Figure 5g,h).

## CD47 promotes CD8+ T cell-mediated immunity to persistent LCMV infection in mice

**Figure 6.**
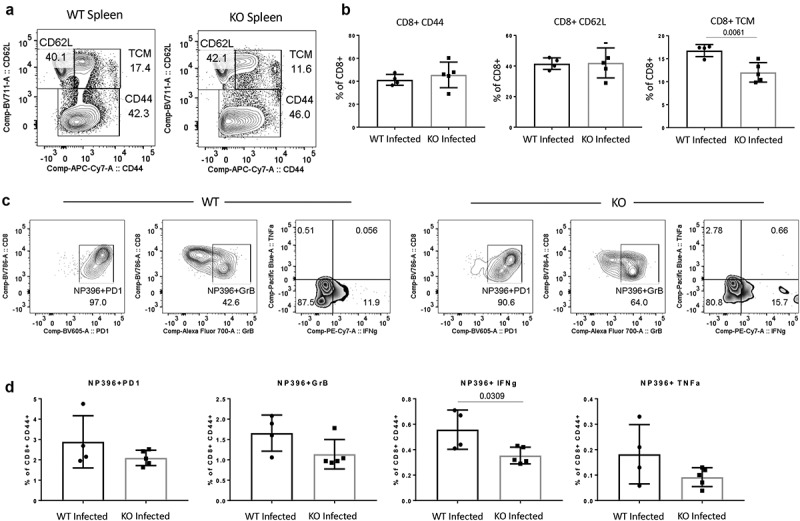
CD47 promotes CD8+ T cell-mediated immunity to persistent LCMV infection in mice.

CD47 was dispensable in the initial CD8+ T cells response to LCMV-Arm infection based on the phenotype on the 8th day of infection. However, when the infection was persistent with LCMV-Cl13, on the 25th day of infection, we observed a significant decrease of CD8+ T cells in *Cd47*^−/−^ mice. Further, immunophenotyping revealed that the limited CD44+ effector phenotype and the elevated CD62L+ naïve phenotype of CD8+ T cells was not CD47 dependent (Figure 6a,b). However, we observed a significant decrease of the central memory (TCM, CD44+ CD6L+) population of CD8+ T cells in *Cd47*^−/−^ mice compared to the WT mice. To precisely phenotype the viral-specific CD8+ T cells, we used LCMV NP396 peptide-specific tetramers. Presence of these cells was evident within the CD44+ effector compartment of CD8+ T cells, with no apparent difference in the frequency between the two groups. Restimulating these cells with LCMV peptide showed a significant decrease of IFNγ accumulation and decreasing trend of PD1, GzmB and TNFα accumulation in *Cd47*^−/−^ mice compared to the WT mice (Figure 6c,d). These data recapitulate the *in vitro* finding of *Cd47*^−/−^ CD8+ T cell phenotype after sustained TCR stimulation.

(a, b) Representative contour plots (values indicate percentage of CD8+ cells) and frequency of naïve (CD44-CD62L+), T central memory (Tcm, CD44+ CD62L+), and T effector memory (CD44+ CD62L-) CD8+ T cells within the spleens of infected mice on the 25th day post LCMV-Cl13 infection. (c, d) Representative contour plots (values indicate percentage of CD44+ cells) and frequency of virus-specific CD8+ T cells within the spleens of infected mice on the 25th day post infection. n = 4 WT mice and 5 *Cd47*-/- mice.

## CD47 deficiency limits CD8+ TILs in B16 melanomas in mice

Earlier, we have found a depletion of effector NK cells in syngeneic B16 tumor bearing *Cd47*^−/−^ mice, which was associated with elevated B16 tumor growth in these mice.^29^ Previous studies treating immunocompetent and T cell-deficient mice bearing immunogenic syngeneic tumors with a CD47-blocking antibody or antisense morpholino combined with tumor irradiation observed tumor regression only in immunocompetent mice but not when CD8^+^ T cells were depleted.^19,21^ These data indicate that an CD8+ T-cell response is required for the antitumor activity of therapeutic CD47 blockade. Restoration of CD47 antisense-dependent tumor ablation by adoptive transfer of tumor-specific CD8 T cells into athymic tumor-bearing mice further established the central role of CD8 T cells.^19^

**Figure 7.**
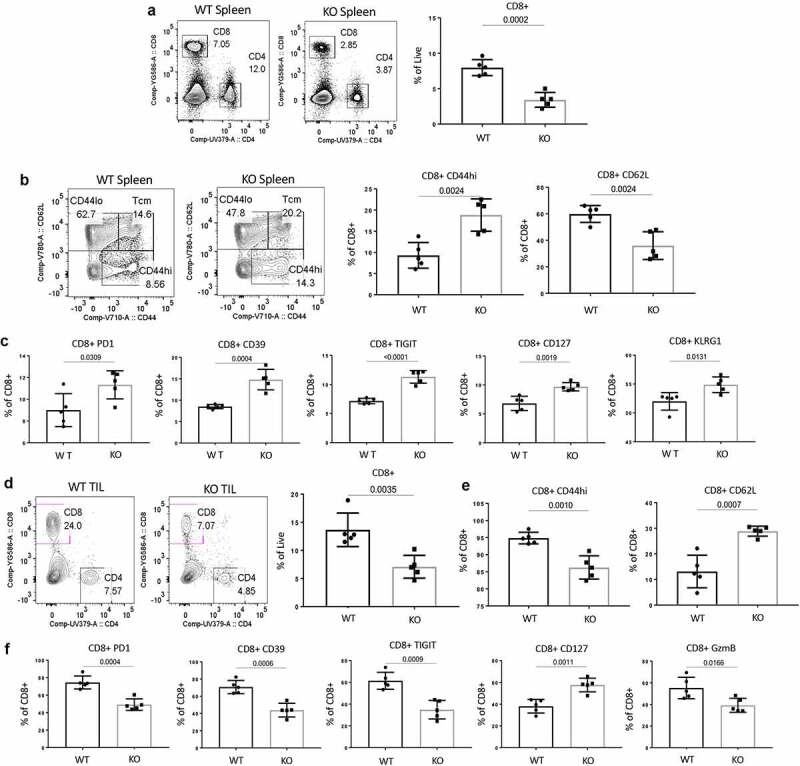
Expedited growth of B16 melanomas in CD47-deficient mice is associated with decreased CD8+ effector T cells.

As observed before, B16 growth was significantly higher in *Cd47*^−/−^ compared to WT mice on day 15th post tumor implantation.^29^ We found splenic CD8+ T cells were significantly decreased in tumor-bearing *Cd47*^−/−^ mice (Figure 7a). However, immunophenotyping of splenic CD8+ T cells revealed that these cells are more CD44+ and less CD62L+ in *Cd47*^−/−^ mice (Figure 7b). As supporting evidence of their elevated immune-reactivity, we found elevated surface expression of PD1, the ectonucleotidase CD39, T cell immunoreceptor with Ig and ITIM domain (TIGIT), CD127, and KLRG1 level in the *Cd47*^−/−^ CD8+ T cells (Figure 7c).

(a) B16F10 tumors were implanted in the hind limbs of WT and *Cd47^−^*^/-^ littermate mice (1 x 10^6^ cells/mouse). On day 15 after tumor implantation, mice were euthanized, and single-cell suspensions of spleens were stained for CD8+ T cells (values indicate percentage of CD3+ cells). (b) Representative contour plots (values indicate percentage of CD8+ cells) and frequency of CD44lo CD62L+ and CD44hi CD62L- CD8+ T cells within the spleens of mice on the 15th day post tumor implantation. (c) Frequency of PD1, CD39, TIGIT, CD127, and KLRG1-positive CD8+ T cells within the spleens of WT and *Cd47^−^*^/-^ (KO) mice on day 15 after tumor implantation. (d) Representative contour plots (values indicate percentage of CD3+ cells) and frequency of CD8+ T cells within the tumor of mice on the 15th day post tumor implantation. (e) Frequency of CD44hi and CD62L+ CD8+ T cells within the tumors of mice on the 15th day post tumor implantation. (f) Frequency of PD1, CD39, TIGIT, CD127, and GzmB-positive CD8+ T cells within the tumors of WT and *Cd47^−^*^/-^ (KO) mice on day 15 after tumor implantation. Representative of two independent experiments, n = 5 mice in each group.

Next, to evaluate the effect of tumor-associated CD8+ T cells, we analyzed the tumor infiltrating lymphocytes (TILs). We found a significant decrease of CD8+ TILs in *Cd47*^−/−^ mice (Figure 7d). Phenotypically, CD8+ TILs of *Cd47*^−/−^ mice showed a significant decrease of CD44+ effector phenotype and a concomitant increase of CD62L+ phenotype (Figure 7e), consistent with our finding in the context of LCMV Cl13 infection. Interestingly in contrary to the splenic CD8+ T cells, CD8+ TILs revealed a significant decrease of surface expression of PD1, CD39, TIGIT as well as intracellular levels of the effector protein GzmB in the *Cd47*^−/−^ mice (Figure 7f).

## Discussion

Although CD47 is an innate as well as adaptive immune checkpoint, mice genetically deficient of *Cd47* are viable and lack an obvious phenotype unless subjected to stress. Therefore, *Cd47*^−/−^ mice have been a successful model to study the role of CD47 in immune homeostasis after introducing experimental infections and tumors. In the present study, we observed no difference in CD8+ T cell development and early activation in *Cd47*^−/−^ mice. Transcripts of some early activated gene clusters defined using pathogen-reactive CD8+ T cells upon immunization in mice differed in 6 h TCR-stimulated WT and *Cd47*^−/−^ CD8+ T cells. Furthermore, TCR-stimulated *Cd47*^−/−^ CD8+ T cells proliferated faster in vitro and had elevated activation and apoptosis transcript signatures. Such phenotype correlates with the early contraction response of pathogen-reactive T cells. Correspondingly, the response of *Cd47*^−/−^ CD8+ T cells after prolonged TCR stimulation exhibited a significant reduction of T cell effector phenotypes as well as elevation of apoptotic cell death. While no apparent difference in the expansion and activation of CD8+ T cells upon acute LCMV infection was observed between WT and *Cd47^−/−^* mice, chronic infection with LCMV Cl13 was elevated in the latter,^28^ which correlated with decreased numbers and effector phenotypes of virus-specific CD8+ T cells. Correspondingly, growth of persistently implanted syngeneic B16 tumors was faster in *Cd47*^−/−^ mice,^29^ which also correlated with the decreasing number and effector phenotypes of tumor-infiltrating CD8+ T cells. Our data clearly shows that CD47 differently regulates CD8+ T cell responses to early and persistence stimulation.

CD47 can play both promotive and protective roles based on the type of infection in mice. During bacterial *E. coli* infection, deficiency of CD47 protected mice from LPS-induced acute lung injury and pneumonia.^62^ *Cd47*^−/−^ mice had reduced parasitemia following infection with *Plasmodium yoelii* malaria parasites and enhanced survival compared to WT mice, which was attributed to enhanced phagocytosis of infected red blood cells (RBCs).^63,64^ In an influenza vaccine study, CD47-deficient mice responded to vaccination with increased titers of virus-specific antibodies and were better protected than WT mice.^65^ The protection mechanism against the viral infection was attributed solely to virus-specific antibodies.^65^ However, chronic LCMV-infected mice exhibit a severe defect in Fcγ-receptor (FcγR)-mediated antibody effector function.^66^ Therefore, similar IgG-mediated protection to LCMV infection is not anticipated in *Cd47*^−/−^ mice. *Candida albicans* fungal infection, on the other hand, showed increased morbidity and mortality of *Cd47*^−/−^ mice due to wider dissemination of infection along with elevated neutrophil, macrophage and CD4 + T cell infiltrates, and increased inflammatory cytokine levels.^67^ Treatment of mice infected with Ebola virus with the function blocking CD47 miap301 adversely affected survival with increased inflammatory cytokine responses, B cell activation, and CTLA4+ CD8+ and CD4 + T cells.

The prolonged T cell-dependent cutaneous inflammation induced by oxazolone in *Cd47^−/−^* and *Thbs1^−/−^* mice relative to the rapid resolution of inflammation in wild type mice, and demonstration of T cell death induced by a CD47-binding peptide from TSP1 provided the first evidence that TSP1/CD47 signaling generally limits T cell activation in the context of inflammation.^15^ In addition, chronic vascular inflammation and risk for atherosclerosis were associated with the disruption of programmed cell removal, and CD47 antibody blockade enhanced macrophage-mediated phagocytosis in atherosclerotic lesions.^68^ Conversely, adeno-associated virus (AAV)-induced hypercholesterolemia and induction of atherosclerosis plaque formation were increased in *Cd47^−/−^* mice.^69^ Studies from our and other labs have established that loss of CD47 causes activation of dendritic cells, T cells and NK cells. NK cell depletion by the treatment with anti-NK1.1 monoclonal antibody, but not CD4/CD8 T cell depletion, equalized atherosclerotic burden, suggesting that NK cells were involved in the enhanced disease in *Cd47*-deficient mice. Other studies have demonstrated that *Cd47* deficiency or CD47-Fc protein treatment limits several inflammatory diseases including colitis,^70^ bacterial induced arthritis,^71^ experimental autoimmune encephalitis,^72^ experimental autoimmune uveitis,^73^ SLE,^74^ and bacterial pneumonia.^62^

Complete loss of CD47 protein expression may also impact the development and differentiation of immune cells. We have shown enhanced differentiation of immature NK cells to mature NK cells in both bone marrow and spleen of *Cd47*^−/−^ mice.^28^ Mature *Cd47*^−/−^ NK cells exhibited increased expression of NK effector and interferon gene signatures and increased proliferative response to interleukin-15. *Cd47*^−/−^ mice showed no defect in their early response to acute LCMV-Arm infection but was moderately impaired in controlling chronic LCMV-Cl13 infection, which was associated with NK cell depletion and loss of NK-effector cytokine and interferon gene expression.^28^ Extending these finding to the role of CD47 in cancer, we observed that melanoma-bearing *Cd47*^−/−^ mice exhibited decreased splenic NK cell numbers, impaired NK-effector protein expression and elevated NK-exhaustion markers.^29^ However, with LCMV Cl13 infection and B16F10 implantation, a significant increase of viral load and tumor growth in *Cd47*^−/−^ mice was observed after a week, indicating subsequent impairment of CD8+ T cell-mediated immune responses.

To evaluate the role of CD47 in T cell development, we compared thymocytes of *Cd47*^−/−^ with littermate and sex-matched WT mice. The development of CD8+ T cells within the thymus as well as early activation (phosphorylation of intracellular tyrosine residues) were not affected in the absence of CD47. Similarly, T cell numbers in thymus and spleen were not altered in mice lacking *Thbs1*.^75^ Previous work indicated that TSP1/CD47 signaling does not regulate thymic maturation of CD4 and CD8 T cells, but it alters T cell differentiation.^67,76,77^ When we stimulated CD8+ T cells with plate-coated anti-CD3 plus soluble anti-CD28 for 48 hours, we saw CD47 deficiency leads to elevated CD69, IL2, and IFNγ expression as well increased CFSE dilution. Earlier works have also reported higher rate of T cell proliferation in the absence of CD47 or TSP1 and CD47-dependent inhibition of activation and proliferation by TSP1.^16,17,76^

These data established an inhibitory role of CD47-mediated TSP1 signaling in T cell activation and proliferation. Increased expression of early activation genes like *Cxcl10, GzmB, IL2*, and *IL2Ra* in *Cd47*^−/−^ CD8+ T cells defined in pathogen-reactive CD8+ T cells suggests the role of CD47 in antigen-specific activation of CD8+ T cells. Moreover, such TCR stimulation significantly enriched the apoptosis gene signature as well as elevated level of phosphatidylserine in *Cd47*^−/−^ CD8+ T cells as detected by annexin V binding. This data suggests increased apoptosis of TCR-stimulated CD8+ T cells in the absence of CD47. With persistent TCR stimulation (anti-CD3 + anti-CD28) for 96 hours *in vitro*, the ability of CD8+ T cells to produce effector molecules such as IFNγ and GzmB was greatly reduced in the absence of CD47. These data clearly suggest that CD47 differentially regulates CD8+ T cell activation and fitness in response to short- versus long-term stimuli.

LCMV infection is an excellent model to study both short- and long-term immune responses of CD8+ T cells.^59^ LCMV-Armstrong (Arm) causes an acute infection that clears within a week of infection, whereas the LCMV clone 13 (Cl13) causes chronic infections that persist for months post-infection.^78^ The dynamics of CD8+ T cell responses during acute and chronic LCMV infection are unique. In response to both acute and chronic LCMV infections, CD8+ T cells respond by rapid expansion followed by a contraction phase. Acute Arm infection is resolved within weeks, and memory T cells are maintained by antigen-independent homeostatic proliferation,^59,79^ while persistent stimuli after chronic Cl13 infection leads to T cell exhaustion.^80,81^ Indeed, T cell exhaustion in persistent viral infection was first reported in chronic LCMV infection,^80,82^ which is now known to occur in humans chronically infected with human immunodeficiency virus (HIV) and hepatitis C virus (HCV).^83^ The key features of exhausted CD8+ T cells under persistent viral stimuli include increased expression of multiple inhibitory receptors, e.g., PD1, CD39, TIGIT, etc., that correlate with cellular dysfunction.^84^ PD1 is selectively upregulated in functionally impaired T cells during chronic LCMV infection, and blockage of this receptor can restore their effector functions.^85,86^

Therefore, to model our *in vitro* observation of CD47-dependent T cell activation in an *in vivo* setting, we chose to use both strains LCMV Arm and Cl13 infection in mice. LCMV-Arm viral titer was significantly high on day 8th post-infection in the serum of *Cd47*^−/−^ mice, indicating that CD47 is necessary for resolving the acute viral infection.^28^ We saw an exponential expansion of CD8+ T cells in the spleens of infected mice; however, no significant difference was observed between CD47-sufficient and deficient groups. The proliferation (Ki67), exhaustion (PD1), and activation (CD44) markers were increased many folds in splenic CD8+ T cells upon infection, comparable between both groups of mice. Therefore, the data suggest that increased acute viral titer in *Cd47*^−/−^ mice sera was due to impairment of factor/s other than CD8+ T cells. Earlier, we reported that effector NK cells were significantly depleted in LCMV Arm with infected *Cd47*^−/−^ mice as early as on day 3 post-infection, which can lead to increased viral titer. Depletion of effector NK population was evident also in LCMV Cl13-infected *Cd47*^−/−^ mice on day 25 post infection.^28^ In a separate experiment, we looked at the T cell compartment of LCMV Cl13-infected mice on day 25 post infection. We observed a significant reduction of CD8+ T cells that correlated with significantly high viral titer in *Cd47*^−/−^ mice. Proliferation of CD8+ T cells was reduced; however, expression of PD1 was high in both WT and *Cd47*^−/−^ mice at that time point. No significant difference in expression of CD39 and TIGIT was found in CD8+ T cells of WT and *Cd47*^−/−^ mice (data not shown). We also observed that the frequency of CD44+ CD62L+ T central memory (Tcm) cells were significantly reduced in *Cd47*^−/−^ mice. When we looked at the virus-specific CD8+ T cells by NP396 tetramer staining, we observed a significant reduction of IFNγ and decreasing trend of GzmB and TNFα effector proteins in *Cd47*^−/−^ CD8+ T cells.

The data clearly indicate that *Cd47*^−/−^ mice have persisting yet functionally compromised CD8+ T cells that cannot control chronic viral propagation as effectively as WT CD8+ T cells do. The presence of persistent activated CD8+ T cells without effector function was first reported in chronic LCMV infection,^80^ which was believed to lead to T cell exhaustion.^87^ Work from the next two decades defined an exhausted T cell (Tex) phenotype. Tex cells have lost their effector functions, have elevated expression of inhibitory receptors, altered epigenetic and transcriptional profiles, and are unable to transition to memory T cells.^88,89^ LCMV acutely resolved infection (e.g., LCMV Armstrong, 53b or low doses of WE) or persistent chronic infection (e.g., docile, high doses of WE or clone 13) models have been the most informative in understanding dynamics of T cell exhaustion.^38,39^ Exhausted T cells are characterized by high expression of inhibitory receptors, poor effector functions, and a unique transcriptional and epigenetic program.^90–93^ Similar T cell exhaustion also occurs in cancer,^94^ and CD8+ T cells display hallmarks of T cell exhaustion in mouse tumor models^95,96^ as well as in human cancers.^90,93,97–99^

We therefore tested the effect of CD47 in CD8+ T cell in responding to implanted syngeneic melanoma in a mouse model. In our earlier studies, we observed elevated growth of B16F10 melanoma tumors in *Cd47*^−/−^ mice, which was partly due to depleted NK cell population.^29^ However, looking at the T cell compartment and on day 15 post tumor implantation, we also observed significant reduction of the CD8+ T cells in the spleen and within the tumor infiltrating lymphocytes (TILs). While CD8+ T cells from spleen showed significant upregulation of CD44+ and concomitant decrease of CD62L+ population in *Cd47*^−/−^ mice, the TILs in these mice showed an opposite pattern of expression. After analyzing the tumor infiltrating CD8+ T cells, we found significant reductions of PD1, CD39, and TIGIT levels in *Cd47*^−/−^ mice. The effector function of these cells has also been compromised as evident by significantly decreased intracellular GzmB. These data suggest that the unique microenvironment of tumors regulates T cell phenotype/function differently in the absence of CD47 than within a lymphoid organ. The presence of CD47 enhanced exhaustion phenotype of CD8+ T cells in the tumor microenvironment. However, the expression of IL-7 receptor (CD127) in both spleen and tumor infiltrating CD8+ T cells was significantly higher in *Cd47*^−/−^ than in WT mice, suggesting early differentiation of effector to central memory T cells in the absence of CD47. Similarly, CD47 inhibited the differentiation of naïve to effector CD4 T cells.^77^ Blocking CD47 by a monoclonal antibody or antisense knockdown, therefore, enhanced the anti-tumor function of CD8+ T cells as reported in multiple studies.^19,21,30^ Depletion of *Cd47*^−/−^ CD8+ T cells in the tumor-bearing mice is consistent with an enhanced contraction and differentiation which is further supported by the expression of differentiated central memory marker CD127. Alternatively, the decreased TILs in the *Cd47*^−/−^ hosts could reflect the function of CD47 to mediate extravasation of T cells.^50^

Cellular energy status is instrumental for coordinated inflammatory responses in T cells. To meet bioenergetic demands associated with vigorous proliferation, acquisition of effector function, and memory formation, T cell undergo dynamic metabolic reprogramming at every stage of this response.^100–102^ Viruses alter the host cell metabolism in order to make optimal conditions for their rapid and efficient replication and spread. Similarly, tumor cells rely on enhanced uptake of important nutrients such as glucose to support metabolic signaling, i.e., aerobic glycolysis (the Warburg effect), a primary pathway of glucose metabolism and its by-products for biosynthetic reactions. Naive T cells rely on oxidative metabolism (OXPHOS) and maintain robust mitochondrial quality control. After activation with co-stimulation (through receptors including CD28), effector T cells are characterized by increased glycolysis and glutaminolysis. Unlike memory T cells where effector T cells attain long-lived stem cell-like properties, if antigenic stimuli sustain, for example chronic viral infection or long-term tumor elimination processes, inhibitory receptors such as PD-1 and CTLA4 can reshape T cell metabolism to reduce effector function and lead to metabolic impairments. Tex cells demonstrate reduced glucose and glutamine metabolism, harbor dysfunctional depolarized mitochondria, and dependence on fatty acid oxidation (FAO). Moreover, mitochondria release proapoptotic factors including ROS and cytochrome-c that activate apoptosis programs in cytolytic lymphocytes, CD8 T cells, and NK cells.^103^ The metabolic adjustments in T cell also can be virus-specific, for example, upon TCR stimulation, HBV-specific CD8 T cells markedly increase the expression of de-novo-synthesized glucose transporter 1 (Glut1) to facilitate glucose uptake and glycolysis, maintained higher levels of PD-1, accompanied by lower expansion and reduced production of IFNγ than CMV-specific CD8 in the same host.^104^ Recent studies on COVID-19 patients showed that elevated glucose levels and glycolysis promote SARS-CoV-2 replication and cytokine production in monocytes through a mitochondrial ROS/hypoxia-inducible factor-1α (HIF-1α)-dependent pathway, resulting in T cell dysfunction and epithelial cell death.^105,106^ Thus, mitochondria serve as a shared platform for metabolism and apoptosis. Previously, we have shown CD47-dependent alterations in multiple mitochondrial metabolites, including basal citrate and citrate synthase levels, were identified in WT versus CD47-deficient Jurkat T cells in the absence and presence of stress induced by ionizing radiation.^26^ Basal mitochondrial oxygen consumption was elevated in CD47-deficient Jurkat T cells, and levels of S-lactoylglutathione, a key metabolite in the detoxification of ROS, were significantly elevated in response to ionizing radiation only in the CD47-deficient cells. Loss of CD47 also results in a defect in mitochondrial metabolism, proton leak, ROS, and increased apoptosis of activated mouse NK cells.^29^ A connection between CD47 and aerobic glycolysis by Enolase 1 (ENO1) has been established.^107^ Shared metabolomic pathways and mitochondrial responses to acute versus persistent infection between cytotoxic NK and CD8+ T cells are expected but yet to be evaluated. The tumor microenvironment (TME), however, itself can diversely affect the effector T cell differentiation and function. Lim et al. reviewed how the TME imposes barriers to the metabolism and activity of tumor infiltrating lymphocytes (TIL).^108^ CD28 co-stimulation can greatly enhance CD8+ TIL metabolism and function through the rescue of T cell glycolysis that supports mitochondrial mass and activity.^109^ CD47 clearly plays a role in CD8+ TIL function. Further studies are required to understand the role of CD47 in T cells co-stimulation and metabolism.

## Supplementary Material

Supplemental Material

## Data Availability

RNA sequencing data of isolated mouse peripheral CD8+ T cells, unstimulated and anti-CD3+CD28 stimulated, is available at the Gene Expression Omnibus (GEO) database under accession number GSE198820. All original code has been deposited at Github and is publicly available as of the date of publication (https://github.com/NIDAP-Community/Loss-of-CD47-alters-CD8-T-cell-activation). All other relevant data are available from the corresponding author directly.
